# The Importance of Systemic Inflammatory Response Syndrome in Diagnosing Occult Infections: A Case Report

**DOI:** 10.7759/cureus.80775

**Published:** 2025-03-18

**Authors:** Jenny Joseph, Syed Alishan Nasir

**Affiliations:** 1 Department of Internal Medicine, Norwalk Hospital, Norwalk, USA

**Keywords:** abscess formation, disseminated bacteremia, infection eradication, methicillin-resistant staphylococcus aureus (mrsa), sirs criteria

## Abstract

Systemic inflammatory response syndrome (SIRS) criteria are among the several screening tools for sepsis. While SIRS has been widely utilized in clinical practice, its reliability and construct validity have come under scrutiny in recent years. Research has established that the quick Sequential Organ Failure Score (qSOFA) and Sequential Organ Failure Assessment (SOFA) criteria serve as better predictors of sepsis-related outcomes. We report a case of a patient who was admitted for diabetic ketoacidosis and atrial fibrillation. Despite being afebrile with stable white blood cell counts, he met SIRS criteria (tachycardic and tachypneic) on admission, which initiated an infectious workup. Subsequent investigations revealed multiple substernal and intramuscular abscesses along with costosternal osteomyelitis resulting in methicillin-resistant staphylococcus aureus (MRSA) bacteremia. The patient underwent surgical source control and recovered after a prolonged course of intravenous antibiotics. This study highlights the importance of timely detection of sepsis and the role of SIRS criteria as a reliable screening tool in this process. Notwithstanding the controversy around the credibility of the SIRS criteria, conceptualized nearly two decades ago, it has proven to have greater sensitivity in the initial diagnosis of sepsis in comparison to other available screening tools. Screening for sepsis using both qSOFA and SIRS criteria concurrently, with an understanding of their limitations, represents best practice.

## Introduction

Sepsis is a life-threatening medical emergency with high mortality and negative long-term implications affecting millions globally. According to the Centers for Disease Control and Prevention (CDC), at least 1.7 million adults in the United States develop sepsis every year out of which at least 350,000 adults died during hospitalization or were discharged to hospice [1]. The global incidence of sepsis is estimated to be almost 30 million annually [2]. One of the vital factors in the successful management of sepsis is early identification of sepsis; therefore, multiple sepsis screening tools were designed to facilitate this process. This includes the systemic inflammatory response syndrome (SIRS) criteria, quick Sequential Organ Failure Score (qSOFA), Sequential Organ Failure Assessment (SOFA) criteria, National Early Warning Score (NEWS), and Modified Early Warning Score (MEWS). Sepsis was first defined in 1991 by a consensus of the American College of Chest Physicians and the Society of Critical Care Medicine as a condition that was primarily caused by the host systemic inflammatory response syndrome [3]. Hence, it was defined as infection with at least two of the four SIRS criteria which included (i) temperature >38°C or <36°C, (ii) heart rate >90/minute, (iii) respiratory rate >20/minute or PA CO_2 _<32 mmHg, (iv) white blood cell count >12,000 per mm^3^ or <4,000 per mm^3^ or >10% immature bands. This marked the beginning of the widespread use of SIRS criteria for sepsis screening in all hospital settings. In recent times, the relevance of SIRS in early sepsis identification has been questioned multiple times. Here we present a case of an elderly man who presented with a seemingly benign picture of diabetic ketoacidosis and atrial fibrillation. Interestingly, he satisfied the SIRS criteria which then prompted an infective workup leading to the diagnosis of MRSA bacteremia with extensive abscesses.

## Case presentation

A 67-year-old male with benign prostatic hyperplasia (BPH) and hypertension presented to the hospital with polyuria, polydipsia, chest pain, and fatigue lasting three weeks. On initial evaluation, the patient was afebrile (36.8°C), tachycardic (132 bpm) with an irregular rhythm, tachypneic (25/min), normotensive (129/95 mmHg), and saturating 98% on room air. Physical examination was significant for a soft tissue mass on his left clavicle. Laboratory studies were remarkable for high anion gap metabolic acidosis with hyperglycemia, ketonuria, and elevated beta-hydroxybutyrate with the absence of leukopenia/leukocytosis/bandemia (Table 1).

**Table 1:** Laboratory results on admission. BUN: blood urea nitrogen

Parameters	Patient values	Reference range
Anion gap	23 mmol/L	8-12 mmol/L
BUN	28 mg/dL	6-23 mg/dL
Creatinine	0.72 mg/dL	0.67-1.23 mg/dL
Glucose	477 mg/dL	70-100 mg/dL
Lactic acid	4.1 mmol/L	0.5-2.2 mmol/L
Bicarbonate	17 mmol/L	22-29 mmol/L
Beta-hydroxybutyrate	1.6 mmol/L	≤0.4 mmol/L
WBC	10.5x10^9^/L	3.5-10 x 10^9^/L
Hemoglobin	12.2 g/dL	13.5-17 g/dL

**Figure 1:**
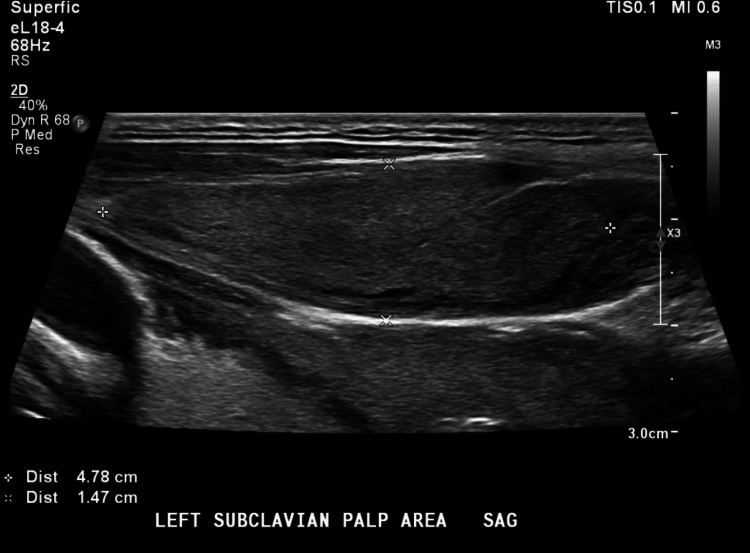
Ultrasound of the neck showing a left supraclavicular abscess.

Additionally, he was also found to be in new-onset atrial fibrillation with rapid ventricular response (RVR). Thus, the patient was admitted for the treatment of diabetic ketoacidosis and atrial fibrillation. Given positive SIRS criteria (tachycardia and tachypnea), an infective work-up was initiated, revealing a positive urinalysis suggestive of UTI. Furthermore, both urine and blood cultures were positive for MRSA. On ultrasound of the neck, a well-circumscribed heterogeneously hypoechoic lesion was identified at the left supraclavicular area which corresponded with the physical examination finding (Figure 1).

**Figure 2:**
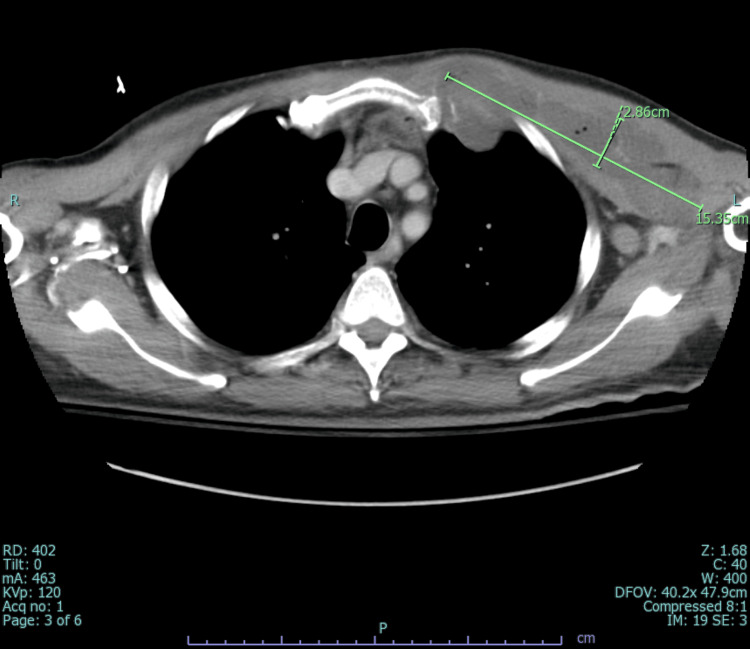
Computed tomography scan (axial view) showing an intramuscular abscess in the left pectoralis muscle.

**Figure 3:**
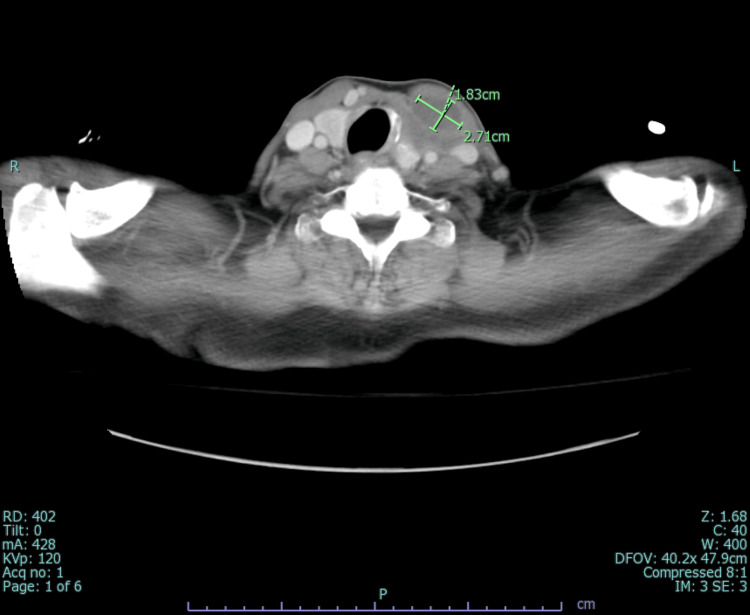
Computed tomography scan (axial view) showing an intramuscular abscess in the left sternocleidomastoid muscle.

An infectious disease physician was consulted for MRSA bacteremia, and treatment with vancomycin and multiple imaging studies were recommended to identify the source. While MRI spine ruled out epidural abscesses and transesophageal echocardiogram (TEE) ruled out infective endocarditis, CT chest/abdomen/pelvis was significant for osteomyelitis of the left first costosternal junction along with multiple seeding intramuscular abscesses involving the left pectoralis (15.4 x 2.9 cm) (Figure 2) and sternocleidomastoid (2.7 x 1.8 x 4.8 cm) (Figure 3).

**Figure 4:**
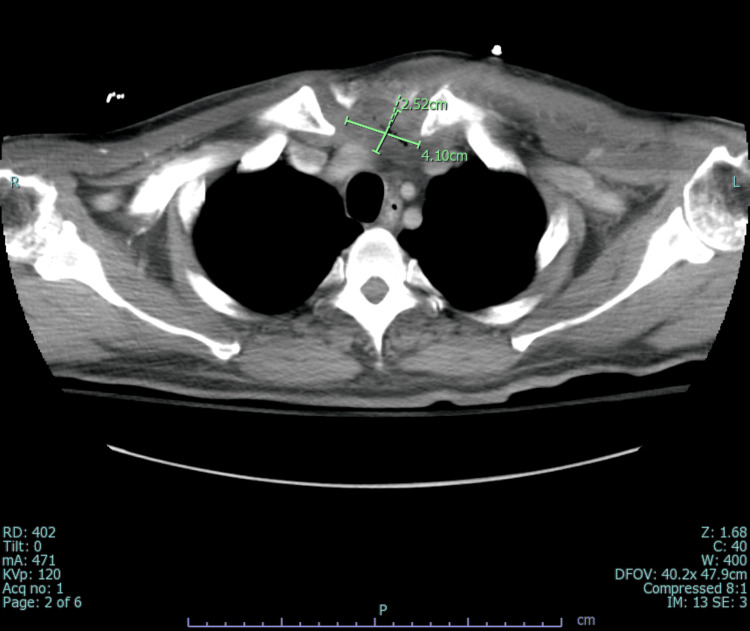
Computed tomography scan (axial view) showing a substernal anterior mediastinal abscess.

**Figure 5:**
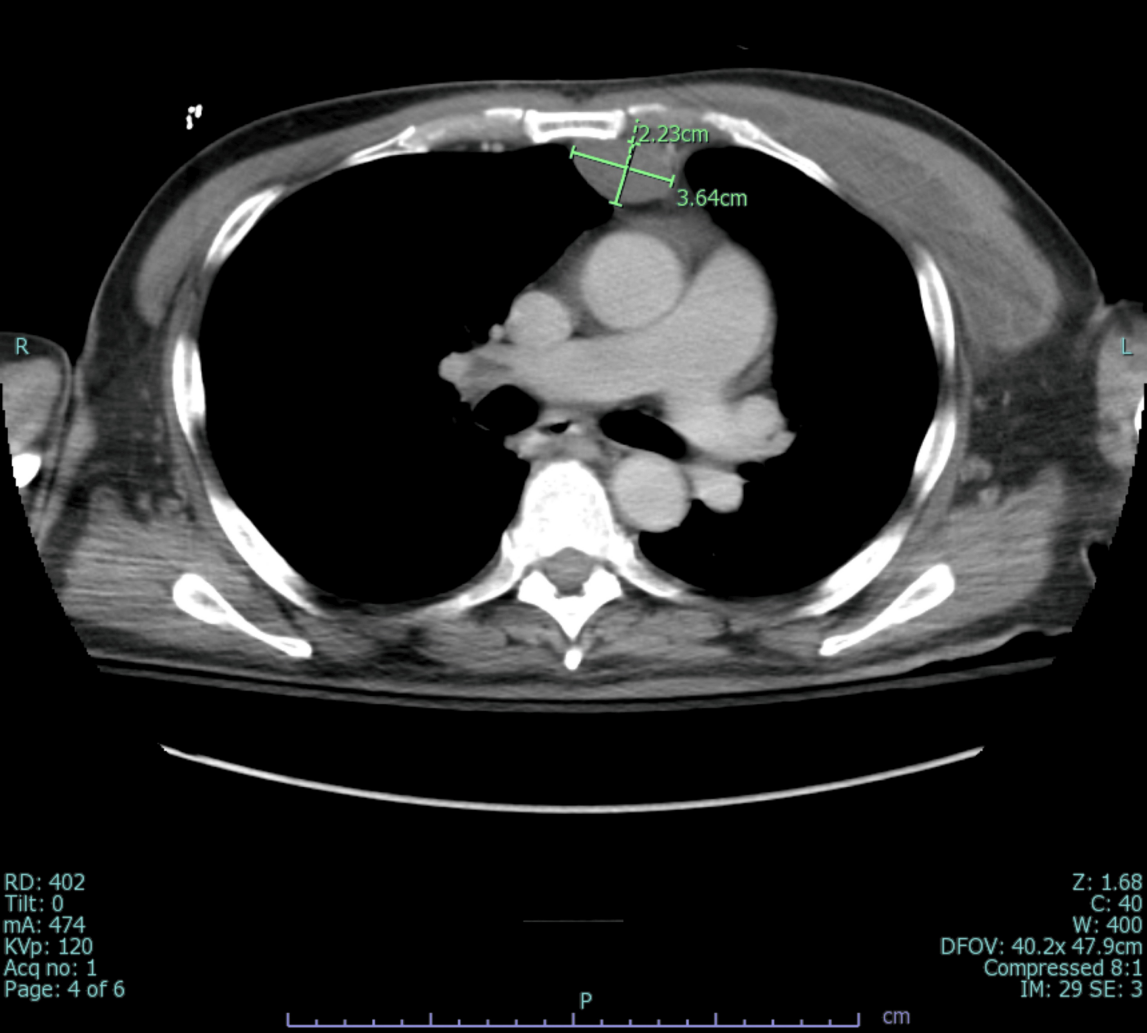
Computed tomography scan (axial view) showing a lower anterior mediastinal abscess.

**Figure 6:**
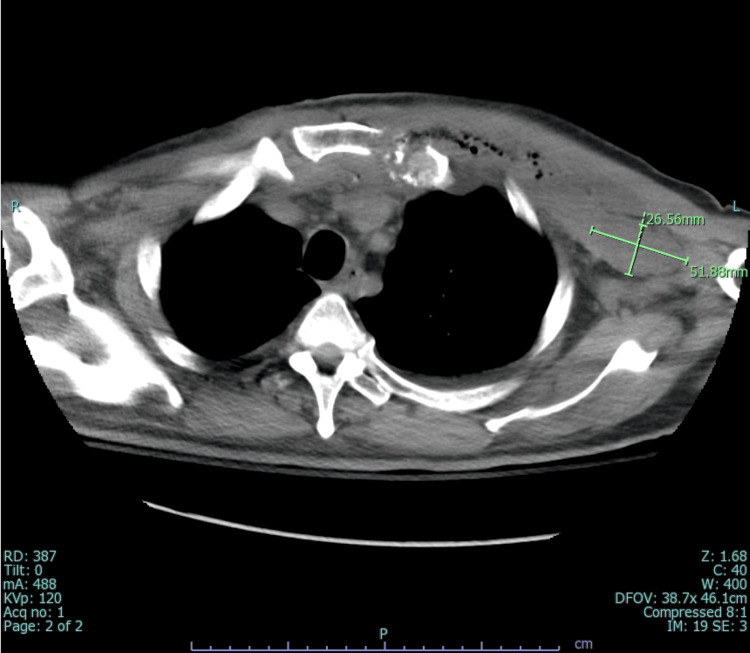
Computed tomography scan (axial view) showing a residual abscess in the left axilla after the first incision and drainage.

It also showed a 4.1 x 2.5 cm abscess in the substernal anterior mediastinum (Figure 4), and an additional lower anterior mediastinal abscess inferiorly measuring 3.6 x 2.2 x 6.0 cm (Figure 5). On the fourth day of admission, the patient underwent source control with drainage of the mediastinal, subpectoral, and left neck abscesses, as well as debridement of the left sternoclavicular and costosternal joints. Repeat CT chest unveiled a residual pocket of abscess material in the left axilla measuring approximately 2.7 x 5.2 cm (Figure 6). Blood cultures were persistently positive for MRSA; however, treatment with vancomycin was continued as failure could not be established without appropriate source control. He underwent repeat incision and drainage (I&D) and washout of new abscesses. With multiple abscesses and continuous positive blood cultures, the risk for other areas of seeding was high, so the patient underwent a whole-body-tagged white cell scan. Post debridement blood cultures stayed positive, accounting for more than seven days of positive MRSA cultures which qualified as persistent MRSA bacteremia. Thus, vancomycin was switched to daptomycin 10 mg/kg and rifampin was added for synergy. Ultimately, blood cultures turned negative, and no new niduses of infection were revealed on the WBC scan. Hence, he was discharged on an extended course of daptomycin and rifampin. The patient was followed through until completion of antibiotics by an infectious diseases physician post-discharge.

## Discussion

The initial definition of sepsis entirely based on the SIRS criteria was challenged by the Sepsis 3 consensus. The task force concluded that an inflammatory response irrespective of the presence of infection could trigger changes in WBC counts, temperature, and heart rate thus reducing the credibility of SIRS criteria [4]. It also pointed out that SIRS criteria could merely be a representation of the body’s appropriate adaptive response to any insult that invokes an inflammatory response. On the other hand, sepsis was characterized by organ dysfunction with a more complex pathophysiology than a simple infection triggering SIRS. Hence in 2021, the Surviving Sepsis Campaign (SSC 2021) redefined sepsis as a life-threatening organ dysfunction secondary to a dysregulated host response which is consistent with the sepsis 3 task force definition [5]. For standardization, an increase in the Sequential Organ Failure Assessment (SOFA) score of two points or more was considered to represent organ dysfunction and was also associated with an in-hospital mortality of greater than 10%.

Among the multiple existing sepsis screening tools, SIRS, SOFA, and qSOFA are predominantly used in the United States whereas NEWS and MEWS are more common in the United Kingdom. Recent literature has increasingly questioned the reliability and clinical relevance of the SIRS criteria in sepsis. Certain studies indicated that nearly half of the hospitalized patients on general medical floors meet SIRS criteria at least once during their admission, rendering it an impractical option for a sepsis screening tool [6]. In 2015, Kaukonen et al. found that one in eight ICU patients with sepsis did not meet the requirement of at least two SIRS criteria [7]. Additionally, they observed a gradual increase in mortality risk with each added SIRS criterion, without a significant jump at the threshold of two criteria, thereby challenging the validity of the established cutoff. Thus, it was determined that the SIRS criteria lacked both discriminant and convergent validity [5].

With the updated definition of sepsis focusing on organ dysfunction, the SOFA score became increasingly preferred. However, the fact that it required laboratory variables like PaO_2_, creatinine, and bilirubin levels restricted its utilization and led to the development of a simplified scale called the qSOFA score (altered mental status, respiratory rate ≥22/min, systolic blood pressure ≤100 mmHg). A positive qSOFA score (greater than or equal to 2) suggested a higher risk of poor outcomes from sepsis. In 2016, Seymour et al. compared the efficacy of SOFA and qSOFA against SIRS and reported that the predictive validity for in-hospital sepsis-related mortality was the greatest for SOFA in the ICU setting and qSOFA in infections outside the ICU [8]. Although the Sepsis 3 task force emphasized utilizing qSOFA and SOFA scores for predicting sepsis-related mortality, they were not intended for initial screening for sepsis.

Numerous studies analyzed the potential use of qSOFA as a screening tool; however, the results were contradictory. While there is a constant debate regarding the relevance of SIRS criteria, a meta-analysis by Fernando et al. highlighted that the SIRS criteria had a higher sensitivity (88.1%) when compared to qSOFA (60.8%), re-emphasizing its superiority as a screening test [9]. On the other hand, a 2019 study by Herwanto et al. concluded that qSOFA outperformed SIRS as a predictor of mortality in sepsis [10]. Several other studies proved that SIRS was a better tool for initial diagnosis, while qSOFA was more well-suited for outcome prediction [11]. As a result, SSC 2021 strongly recommended against using qSOFA compared with SIRS, NEWS, or MEWS as a single screening tool for sepsis or septic shock due to its low sensitivity [5]. It also advised that both SIRS and qSOFA must be applied with consideration of their respective limitations.

In the present case, the patient met SIRS criteria (tachycardia and tachypnea) on admission which led to an infectious work-up. This investigation revealed multiple MRSA abscesses that caused persistent MRSA bacteremia, with blood cultures remaining positive for more than seven days, necessitating prolonged intravenous antibiotic therapy. However, despite the extensive nature of the infection, the patient did not have a positive qSOFA score upon admission. While several studies have criticized the sensitivity of SIRS criteria in the early detection of sepsis, none have identified a more sensitive screening tool.

## Conclusions

Early identification of sepsis is crucial for effective management. While newer scoring systems such as qSOFA and SOFA have proven to be better predictors of mortality outcomes in sepsis, they have not surpassed SIRS criteria as a reliable screening tool for sepsis. In some clinical scenarios, such as with the present patient, individuals may meet SIRS criteria despite a negative qSOFA score, yet still have an underlying infectious process. This establishes the continued relevance of SIRS criteria in current medical practice and reiterates that failing to meet two or more qSOFA or SOFA criteria should not lead to a deferral of prompt investigation and treatment. It is essential to account for the limitations of scoring tests and apply them appropriately based on clinical judgment. This case also emphasizes the SSC 2021 recommendation against using qSOFA as a single screening tool in comparison to SIRS for the identification of sepsis or septic shock.

## References

[1] Rhee C, Dantes R, Epstein L (2017). Incidence and trends of sepsis in US hospitals using clinical vs claims data, 2009-2014. JAMA.

[2] Fleischmann C, Scherag A, Adhikari NK (2016). Assessment of global incidence and mortality of hospital-treated sepsis. Current estimates and limitations. Am J Respir Crit Care Med.

[3] Bone RC, Balk RA, Cerra FB (1992). Definitions for sepsis and organ failure and guidelines for the use of innovative therapies in sepsis. Chest.

[4] Singer M, Deutschman CS, Seymour CW (2016). The third international consensus definitions for sepsis and septic shock (Sepsis-3). JAMA.

[5] Evans L, Rhodes A, Alhazzani W (2021). Surviving sepsis campaign: international guidelines for management of sepsis and septic shock 2021. Intensive Care Med.

[6] Churpek MM, Zadravecz FJ, Winslow C, Howell MD, Edelson DP (2015). Incidence and prognostic value of the systemic inflammatory response syndrome and organ dysfunctions in ward patients. Am J Respir Crit Care Med.

[7] Kaukonen KM, Bailey M, Pilcher D, Cooper DJ, Bellomo R (2015). Systemic inflammatory response syndrome criteria in defining severe sepsis. N Engl J Med.

[8] Seymour CW, Liu VX, Iwashyna TJ (2016). Assessment of clinical criteria for sepsis: for the third international consensus definitions for sepsis and septic shock (Sepsis-3). JAMA.

[9] Fernando SM, Tran A, Taljaard M, Cheng W, Rochwerg B, Seely AJ, Perry JJ (2018). Prognostic accuracy of the quick sequential organ failure assessment for mortality in patients with suspected infection: a systematic review and meta-analysis. Ann Intern Med.

[10] Herwanto V, Shetty A, Nalos M, Chakraborty M, McLean A, Eslick GD, Tang B (2019). Accuracy of quick sequential organ failure assessment score to predict sepsis mortality in 121 studies including 1,716,017 individuals: a systematic review and meta-analysis. Crit Care Explor.

[11] Serafim R, Gomes JA, Salluh J, Póvoa P (2018). A comparison of the quick-SOFA and systemic inflammatory response syndrome criteria for the diagnosis of sepsis and prediction of mortality: a systematic review and meta-analysis. Chest.

